# Establishing robotic bariatric surgery at an academic tertiary hospital: a learning curve analysis for totally robotic Roux-en-Y gastric bypass

**DOI:** 10.1007/s11701-022-01454-1

**Published:** 2022-08-22

**Authors:** Anne Kauffels, Martin Reichert, Ingolf Askevold, Anna Bender, Andreas Hecker, Winfried Padberg, Thilo Sprenger

**Affiliations:** Department of General, Visceral, Thoracic, Transplant and Pediatric Surgery, University Hospital of Giessen, Rudolf- Buchheim-Str. 7, 35392 Giessen, Germany

**Keywords:** Robotic surgery, Roux-en-Y gastric bypass, Learning curve

## Abstract

The use of robotic systems in bariatric surgery has constantly increased over the last years. However, beside its technical advantages in morbidly obese patients the conclusive role of robotics in bariatric and metabolic surgery is still under controversial debate. This is an analysis of prospectively collected data of consecutive patients undergoing fully robotic Roux-en-Y gastric bypasses (TR-RYGB) during the first year after implementation of a robotic bariatric program at a tertiary university hospital. All patients were operated by a previously untrained robotic but experienced laparoscopic bariatric surgeon using the daVinci Xi system (Intuitive Surgical, Sarl). Data recording included patient characteristics, operative and functional outcomes, complications and learning curves for surgeon and assistants. In total, 80 patients underwent primary or revisional robotic bariatric surgery. Seventy-two patients (90%) received a TR-RYGB. There were no major complications, re-interventions or readmissions. The overall complication rate was 2.5% (Clavien–Dindo grade I and II). The overall operation time was 140.7 ± 24.6 min and decreased significantly from the first to the last decade of procedures (procedure 1–10: 171.2 ± 26.3 min versus procedure 63–72: 116.0 ± 10.9 min, *p* < 0.0001). A stabilization of the learning curve was observed after 30 procedures for the surgeon and after five procedures for the bedside assistant. With immediate effect, TR-RYGB is a safe procedure with low complication rates for an experienced laparoscopic bariatric surgeon without prior robotic skills. Learning curves are steep and operation times can be effectively decreased by increasing the experience of the surgeon.

## Introduction

The Roux-en-Y gastric bypass (RYGB) is one of the most commonly performed bariatric operations and globally one of the standard procedures particularly in case of concomitant type-2 diabetes and/or gastroesophageal reflux [1, 2]. The conventional laparoscopic approach for RYGB (L-RYGB) is well established and can be performed safely and with low complication rates after overcoming the learning curve of at least 100 [3] up to 500 cases [4]. However, L-RYGB comprises immanent limitations including the counterintuitive movement of instruments and the restricted degree of freedom and instrumental movement especially against the backdrop of the excessive counterforce of the obese abdominal wall. The robotic approach for RYGB (R-RYGB) in morbidly obese patients is thus regarded as a promising tool to overcome the limitations of standard laparoscopy. Robotic surgery ensures a stable 3-dimensional high definition vision, control of three robotic arms including a binocular camera, precise intracorporal movements due to highly flexible instruments with an extended range of motion. Particularly in obese patients the remote center technology can help to reduce lever movements and minimize traumatization of the trocar-sites within the abdominal wall.

Nevertheless, the role of R-RYGB is still controversial. Beside tendentially higher costs for the robotic procedure, some smaller, predominately single-center studies have reported higher complication rates in R-RYGB [5, 6]. Other studies showed lower complication rates and less revisional procedures but longer operation times for R-RYGB compared to L-RYGB [7–9]. Along with the increasing acceptance for the robotic approach in bariatric surgery the number and quality of studies in this field has distinctly advanced throughout the last few years. Data from the Metabolic and Bariatric Surgery Accreditation Quality and Improvement Program (MBSAQIP) database consistently show longer operation times for R-RYGB but simultaneously an improvement in perioperative outcomes with lower overall complication rates and decreased length of hospital stay (LOS) compared to L-RYGB [10, 11]. An evaluation of almost 80,000 patients recorded in the MBSAQIP database who underwent RYGB in 2015 and 2016 demonstrated lower mortality, less bleeding complications, transfusion requirements and wound infections for the R-RYGB cohort [12]. However, despite the still inconsistent conclusions regarding its surgical value as well as its economic justification the use of robotics is globally increasing both in primary [13] and in revisional bariatric surgery [14–16].

Here, we report our single-institution, single-surgeon results, intra and postoperative complications, morbidity data as well as learning curve analyses after establishing a robotic bariatric program with totally robotic RYGB (TR-RYGB) using the daVinci Xi system (Intuitive Surgical, Sarl) including robotic stapling techniques and hand-sewn gastrojejunostomy in 80 morbidly obese (WHO class III) patients. Given, that there are still conflicting data about safety, complication rates and learning curves for R-RYGB [17–20] the major aim of our study was to investigate safety, procedural learning efficiency and short-term outcome data after implementation of a TR-RYGB technique performed by an experienced laparoscopic bariatric surgeon.

## Patients and methods

All bariatric operations at our center were performed by a certified bariatric surgeon and have been prospectively recorded. Indications for bariatric surgery were based on multidisciplinary recommendations and according to the national guidelines.

The daVinci Xi system was acquired in 2019 at our institution and has mainly been used for urologic and colorectal operations. With implementation of the robotic bariatric program, we decided to exclusively perform (primary and revisional) bypass procedures (with RYGB as our institutional gold standard for primary surgery). Due to presumably limited benefit for short and long-term outcome parameters of the robotic approach, the sleeve gastrectomy (SG) was determined to furthermore be exclusively performed laparoscopically as it is comparably uncomplex, requires less intraoperative dexterity and frequently takes 30–45 min at our center.

From 04/2021 to 04/2022 eighty patients underwent robotic bariatric surgery in our institution. 72 patients (90%) had primary TR-RYGB, 5 patients (6%) had revisional surgery after primary L-RYGB and three patients had intraoperative conversion to TR-SG due to massive interenteric adhesions (two patients) and histologically proven liver cirrhosis based on non-alcoholic steatohepatitis (NASH) (one patient).

A retrospective analysis of prospectively collected data was performed including patient sex, age, weight, body mass index (BMI), American Society of Anaesthesiologists (ASA) classification, Edmonton Obesity Staging System (EOSS), metabolic disorders and history of prior abdominal surgery. Operation time (as the continuum from first incision to skin closure, including robot docking and console time), blood loss, intraoperative as well as postoperative complications according to the Clavien–Dindo classification [21] including anastomotic insufficiency, stenosis, re-operation, surgical site infection rates after 30 days and, if applicable, three, six and 12 months respectively, length of hospital stay (LOS) and readmission rates were assessed.

### Surgical and anastomosis technique

Generally, the technique for TR-RYGB was based on our technique for L-RYGB except for the gastrojejunostomy, which is usually performed as side-to-side anastomosis with a linear stapler in L-RYGB and has been replaced by a hand-sewn two-layer functional end-to-end gastrojejunostomy with a maximal diameter of 1.5 cm in the robotic approach.

TR-RYGB was performed in reverse Trendelenburg position (15°). Three 8 mm and one 12 mm daVinci trocars were positioned in a horizontal line 15 cm below the xiphoid. Another 12 mm assist-trocar in the upper right quadrant or a subxiphoidal Nathason retractor were placed for retraction of the left liver segments. Due to the relatively high mean BMI and the incidence of hepatic steatosis in our cohort, we preferred the additional trocar and the Endo Paddle Retract^®^ (Medtronic, Minneapolis, USA) for liver retraction. We used a fenestrated bipolar forceps and the 60 mm SureForm^®^ stapling device (arm 1), a 30°-angle endoscope (arm 2), the Vessel Sealer Extend® and a needle driver (arm 3) and the tip-up fenestrated grasper (arm 4).

A rather small (30-40 ml) gastric pouch was calibrated on a 36-Fr. orogastric tube by robotic stapling. The biliopancreatic limb of 100 cm jejunum was measured and the jejunal loop was positioned upward in a tension-free antecolic antegastric fashion. A robotic hand-sewn two-layer end-to-side (functional end-to-end) gastrojejunostomy with a maximal width of 1.5 cm was performed with running sutures of 2–0 PDS Stratafix^®^ (Ethicon, Cincinnati, OH, USA). The biliopancreatic limb was transected in straight proximity to the gastrojejunostomy and a 150 cm alimentary limb was measured and a stapled 60 mm side-to-side jejunojejunostomy was created with the SureForm^®^ stapler. A methylene-blue test was performed routinely to check for leakages of the gastrojejunostomy. Placement of drainage was obsolete in all cases.

### Statistical analysis

Statistical analyses were performed using GraphPad Prism (Version 9, GraphPad Software, San Diego, CA, USA). For descriptive statistics, group comparisons of continuous variables were performed either by MWU test for two-group comparisons or by Kruskall-Wallis test for global effects and, if applicable, followed by Dunn’s multiple comparisons test of each group with the control group (i.e. patients 61–72). Bars in the boxplots depict median, whiskers indicate the minimum to maximum range, the boxes extend from the 25th to 75th percentiles and indicate the interquartile range. Simple linear regression analysis was used to predict significant dependencies between the procedures performed during the learning curve phase and relevant variables of perioperative outcomes. Results are given as r_2_ and respective significances.

To determine statistical dependences between operation time and relevant patient and procedure characteristics as well as with the robotic experiences of the surgical assistant (stratified by ≥ 5, ≥ 8 and ≥ 10 robotic assists) independently from the learning curve assessment of the console surgeon, Spearman’s Rho rank correlation was used. Results are given as the Spearman’s rank correlation coefficient (r_sp_) and respective significances.

*p* values ≤ 0.05 indicate statistical significance. Because of the exploratory character of the study no adjustments of *p* values were performed.

## Results

### Patients characteristics

In total, 80 patients underwent primary or revisional totally robotic bariatric procedures with the daVinci Xi system after its implementation between 04/ 2021 and 04/2022. The majority of patients received TR-RYGB (*n* = 72; 90%). Four patients had robotic revisional surgery after earlier L-RYGB with pouch-resizing, resection and recreation of a hand-sewn gastrojejunostomy due to massive dilatation with severe symptoms of dumping (*n* = 3) and due to chronic anastomotic-gastric fistula with insufficient weight loss (*n* = 1). One patient had robotic conversion of One-Anastomosis Gastric Bypass (OAGB) to RYGB due to therapy-refractory bile reflux. Three patients initially intended to receive TR-RYGB were intraoperatively converted to TR-SG due to massive adhesions (*n* = 2) or histologically proven NASH-based liver cirrhosis (*n* = 1).

For statistical evaluation only patients with TR-RYGB (*n* = 72) were considered to obtain comparability in regard of the assessed parameters (e.g. operation time). Nevertheless, the revisional procedures contributed to individual experience and learning curves. Clinical characteristics are summarized in Table 1.

**Table 1:** Clinicopathological Findings of the Total and TR-RYGB-Cohort

Feature	Number of patients undergoing robotic bariatric surgery *n* = 80	%^a^	Number of patients undergoing TR-RYGB *n* = 72	%
Gender				
Male	21	26	19	26
Female	59	74	53	74
Age (years)				
Mean	43.65		43.67	
SD	10,85		10.79	
Body mass index (kg/m^2^)				
Mean	49.93		50.54	
SD	6,25		5.37	
Body weight (kg)				
Mean	140.7		142.0	
SD	22.3		21.26	
Diabetes mellitus type II				
Yes	32	40	32	44
No	48	60	40	56
Prior abdominal surgery				
Yes	47	59	39	54
No	33	41	33	46
Surgical procedure				
Totally-robotic RYGB	72	90	72	100
Robotic sleeve gastrectomy	3	4	0	0
Revisional Surgery	5	6	0	0
Simultaneous additional surgical procedure(s)				
Yes	22	27	17	24
No	58	73	55	76
Length of hospital stay (days)				
Mean	2.18		2.19	
SD	0.74		0.77	
EOSS (Edmonton obesity staging system) classification				
0	0	0	0	0
1	0	0	0	0
2	71	89	63	87
3	9	11	9	13
4	0	0	0	0
Surgical complications (according Clavien–Dindo)				
0	78	97	70	97
1	1	1	1	1
2	0	0	0	0
3 a	1	1	1	1
b	0	0	0	0
4 a	0	0	0	0
b	0	0	0	0
5	0	0	0	0

^a^Because of rounding not all percentages might result in 100

Mean preoperative body weight in the 72 patients who underwent TR-RYGB was 142.0 ± 21.2 kg and mean BMI was 50.5 ± 5.4 kg/m^2^. Thirty patients (41.6%) had type-2 diabetes and 39 patients (54.2%) had prior (open or laparoscopic) abdominal surgery.

Additional operative procedures were performed in 17 patients undergoing TR-RYGB (24%). Significant adhesiolysis (either laparoscopically or robotically) had to be carried out in two patients (2.7%). Four patients (5.6%) underwent simultaneous robotic cholecystectomy due to symptomatic cholecystolithiasis. Seven patients (9.7%) had evidence of hiatal hernia which was closed by posterior hiatoplasty. Two patients (2.7%) had large (> 10 cm) Morgagni hernia, which had to be closed using mesh repair. Finally, two patients (2.7%) had evidence of histologically proven gastrointenstinal stromal tumors (GIST) as incidental finding und underwent partial resection of the remnant stomach.

### Operation times and learning curves of the surgeon and first assistant

The mean total operation time for TR-RYGB was 140.7 ± 24.6 min over all patients and 149.3 ± 22.4 min in patients with additional operative procedures (e.g. hiatal hernia repair or cholecystectomy). Operation time did not significantly differ between TR-RYGB and TR-RYGB combined with additional procedures although there was a discernible trend for longer operation times in the latter (*p* = 0.059).

**Fig. 1:**
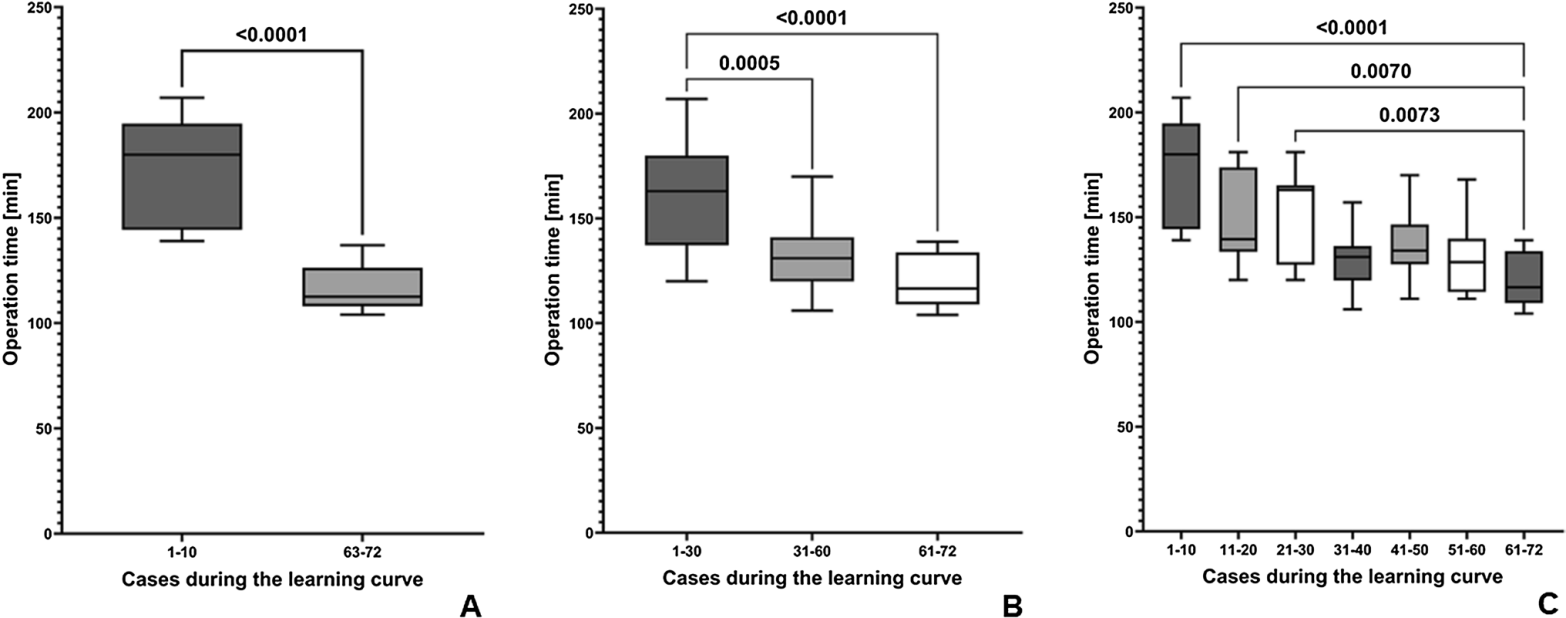
Analysis of operation times during the learning curve. Operation times significantly decreased within the first year after implementation of TR-RYGB and stabilized after 30 procedures. **A** Comparison of procedure 1–10 with 63–72. **B** Comparison of procedure 1–30 and 31–60 and 61–72, respectively. **C** Comparison of each decade of procedures with the last decade of procedures

**Fig. 2:**
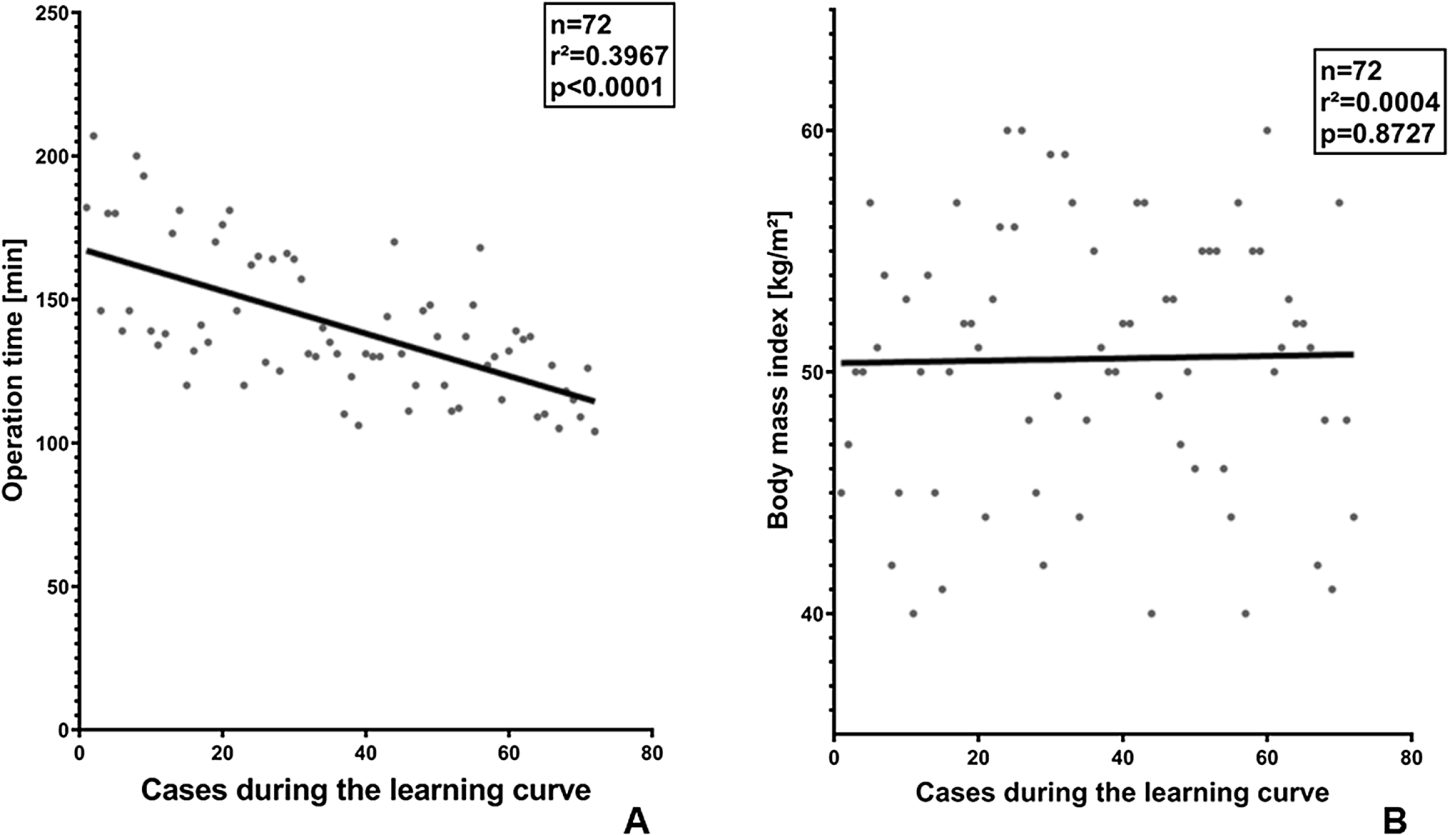
Regression analysis of learning curve determinants. **A** Learning curve of an experienced laparoscopic bariatric surgeon without prior robotic skills over the first year after implementation of TR-RYGB. **B** Development of the body mass index (BMI) of patients undergoing TR-RYGB over the learning curve

With increasing number of procedural exposition and robotic experience, the operation time decreased significantly from 160.6 ± 26.1 min (first 20 TR-RYGBs) to 134.4 ± 15.6 min (procedures 53–72; *p* = 0.0005) or 171.2 ± 26.3 min (first 10 TR-RYGBs) to 116.0 ± 10.9 min (procedures 63–72; *p* < 0.0001), respectively. We observed a general stabilization of the mean operation time after 30 TR-RYGBs with an ongoing and continuous but less clear improvement thereafter (Fig. 1). In regression analysis a significant decrease of operation times could be objectified (*r*^2^ = 0.3967; *p* < 0.0001) over the time (Fig. 2A).

Within the 72 TR-RYGB procedures 12 surgeons without prior robotic experience acted as first (bedside) assistant. Their case load volume ranged from one to 34 assistances after the one year period. In correlation analyses, we observed a trend to shorter operation times when the assistant had a case load volume of ≥ 5 assistant procedures (*r*_sp_ = -0.201; *p* = 0.09). By increasing number of assistances beyond five procedures there was no further effect on operation time (*n* ≥ 8 assists: *r*_sp_ = -0.111, *p* = 0,353; *n* ≥ 10 assists: *r*_sp_ = − 0.026, *p* = 0.831) indicating a rather fast learning gain for bedside assistants under the terms of fully robotic procedures.

No significant correlation was observed between operation time and patient sex, weight or BMI (Fig. 2B), respectively.

### Blood loss and intraoperative complications

Intraoperative blood loss was minimal (≤ 30 ml) in all patients. There was no significant correlation with patient sex, weight and BMI. Blood loss did not significantly change within the study period or over the learning curve (*p* = 0.7).

There were no intraoperative surgical complications and no conversions to either laparoscopic or open surgery. One patient had evidence of anaphylactic shock during operation presumably due to perioperative single-shot antibiotics which made temporarily medical circulatory support and antihistaminics and prednisolone therapy necessary, and was, fully recovered, able to be transferred to the normal ward postoperatively.

### Postoperative complications and length of hospital stay (LOS)

In total, two complications (2.5%) according to the Clavien–Dindo classification occurred. One patient had postoperative bleeding with intraperitoneal hematoma from a 12 mm assist-trocar site (Clavien–Dindo II). One patient undergoing simultaneous cholecystectomy had superficial wound infection after extension of the assist-trocar site access was necessary for removal of the stone-filled gallbladder (Clavien–Dindo I).

No anastomotic leakage occurred. No surgical revision or re-operation was necessary. There was no readmission within 30 days or within the further follow-up so far. No intraluminal hemorrhage occurred. Postoperative anastomotic stenosis, dys- or odynophagia has not been observed within in the follow-up.

General major perioperative complications (thrombosis, pulmonary embolism, pneumonia) did neither occur within 30 days after operation nor within the further follow-up so far. One patient presented with a self-limiting, presumably drug-induced postoperative thrombopenia after perioperative antibiotic prophylaxis without any clinical consequences.

The average LOS was 2.2 ± 0.8 days. There was no significant change of LOS within the study period or over the learning curve (*p* = 0.869).

## Discussion

The role of robotics in bariatric surgery is under ongoing debate and still discussed controversially. Nevertheless, the worldwide use of robotic approaches for bariatric procedures has constantly increased over the last years despite potentially higher costs and longer operation times compared to laparoscopic procedures [22]. In our bariatric center, L-RYGB is well established and frequently performed with low morbidity and complication rates. However, TR-RYGB with hand-sewn gastrojejunostomy might lead to less mid- and long-term anastomotic complications (stenosis or dilatation) with decreased risk for early or late dumping syndrome and necessity for anastomotic revision compared to L-RYGB with stapled gastrojejunostomy. Moreover, to selected patients (super-obesity with BMI > 50 kg/m^2^, revisional surgery) the robotic platform might be particularly beneficial due to the incomparably broader range of motions and its ergonomic advantages [23].

We report on our initial results one year after establishing the institutional bariatric robotic program. In our experience, primary and revisional TR-RYGB with hand-sewn anastomosis is ab initio a safe procedure with low complication rates and a relatively steep learning curve of 30 cases when performed by an experienced laparoscopic bariatric surgeon. Except for the gastrojejunostomy, which is performed as a handsewn end-to-side (functional end-to-end) anastomosis with a diameter of < 1.5 cm in the robotic approach and as a linear-stapled side-to side anastomosis in the laparoscopic approach, we generally transferred our laparoscopic technique and the sequence of the individual surgical steps to the robotic platform.

In contrast to our results, Benziri et al. [5] reported on significantly higher complication rates after total robotic RYGB with hand-sewn gastrojejunostomy. Although the mean operation time (130 min) was comparable to ours, surgical complications occurred in 13% of patients causing increased LOS (9.3 vs. 6.7 days) in those patients who underwent TR-RYGB compared to L-RYGB. Other studies specify the range of anastomotic complications after robotic hand-sewn gastrojejunostomy in detail. Leaks appeared in 7.5% and invariably occurred at the pouch level after TR-RYGB and interestingly not at the anastomotic site presumably indicating basic problems with the creation of the pouch or with stapling-devices or stapling techniques [6]. A study comparing TR-RYGB with hand-sewn gastrojejunostomy and different laparoscopic anastomotic techniques (circular- vs linear-stapled), revealed longer operation times (mean > 200 min) but significantly shorter LOS, lower readmission rates and lesser mid- and long-term anastomotic complications such as strictures of the gastrojejunostomy for the robotic approach [24]. Roriz-Silva et. al [25] compared L-RYBG and R-RYGB after overcoming the respective learning curves and found significantly longer operation times (150.7 min) and more leakages but significantly shorter LOS after R-RYGB.

We had neither anastomotic leakages nor symptomatic anastomotic strictures or intervention-requiring dilatations after TR-RYGB. Handsewn gastrojejunostomy might promise lesser rates of medium and long-term anastomotic problems such as stenoses and/or dilatations compared to stapled anastomoses although a clear effect of the anastomotic technique on weight-loss efficiency or remission of comorbidities could not be shown so far [26, 27].

Mean operation time for TR-RYGB (140.7 ± 24.6 min) over the entire learning period in our series was comparably low and constantly stabilized after overcoming the learning curve of about 30 procedures. Nevertheless, even after 60 TR-RYGB procedures we observed a slight but, however, present decrease of operation times to 116 min ± 11 min. In an early study from 2008, Hubens et al. revealed comparable learning curves of 35 cases. Their overall mean operation times were 212 min and could significantly be decreased to 127 min after 35 procedures. Remarkably, they reported conversion rates of 9% to laparoscopic and 11% to open surgery [28].

Taken into consideration that a hand-sewn double-layer gastrojejunostomy is more time-consuming than a robotically stapled linear side-to-side anastomosis, operation times can only be compared under the aspect of the respective anastomotic technique. As to our study, operation times could further be diminished by adjusting the technique from a handsewn to a stapled gastrojejunostomy. However, since this is the report of our first experience after implementing TR-RYGB, the significance of the gastrojejunal anastomotic technique and its impact on short- and long-term outcomes remains to be assessed.

**Fig. 3:**
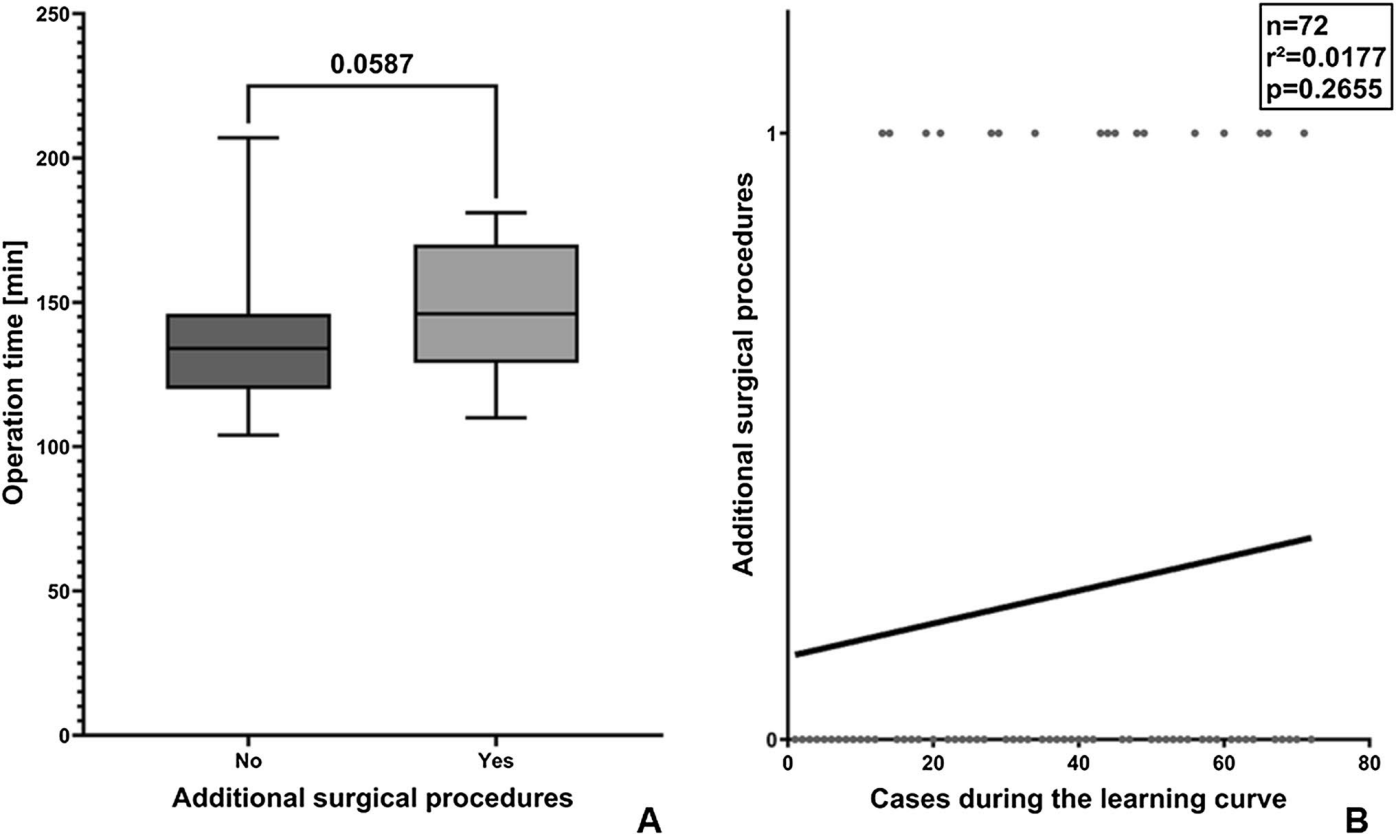
Additional surgical procedures during the study period. **A** Additional surgical procedures combined with TR-RYGB did not significantly affect operation times. **B** Additional surgical procedures combined with TR-RYGB increased over the study period. The missing statistical effect of time-consuming additional procedures (cholecystectomy, closure of hiatal or morgagni hernia) on total operation times is explainable by shorter operation times for the intrinsic TR-RYGB procedure in the later study period

Interestingly, additional procedures (hiatal or morgagni hernia closure or cholecystectomy) did not significantly extend operation times. This might be explainable by the fact that additional procedures were performed predominantly in the later period of this series when operation times for intrinsic TR-RYGB procedures were significantly lower (Fig. 3).

Another considerable point is the role of the (bedside) assistant in TR-RYGB. We observed a very steep learning curve of five procedures after which additional experience of the assistant had no further impact on operation times. Other studies demonstrated a significant and independent influence of the assistant case load on operation time and complications [29]. This discrepancy might be explainable by the minor involvement of the assistant in specific surgical steps under terms of TR-RYGB compared to L-RYGB or R-RYGB with external (non-robotic) stapling by the assistant in that era. However, the intraoperative management of the patient cart and the instrument change can safely and efficiently be performed by the assistant after overcoming a very short learning curve. This finding might contribute to the general discussion of staff- and cost-effectiveness of robotic bariatric surgery to the effect that complex surgical procedures as RYGB are performable as single-surgeon-procedures with assistance by paramedical staff.

In our study, 46 patients (64%) undergoing TR-RYGB had super-obesity with BMI ≥ 50 kg/m^2^ (50–60 kg/m^2^). Nevertheless, there has been no significant correlation between the BMI and the development of complications, longer operation time or LOS indicating that the robotic platform might compensate higher body weight and higher BMI or even level up patients with super-obesity to those with BMI < 50 kg/m^2^ regarding individual operative preconditions and risks.

This study has various limitations. Here, we present our initial results of TR-RYGB of the first year after its implementation. The intention was to demonstrate our experiences and potential pitfalls when “switching” from the laparoscopic to the robotic approach while maintaining the general surgical strategy and procedural steps. With our first results, we did not intend to compare TR-RYGB with L-RYGB. Naturally, we simultaneously performed L-RYGB within the study period. Patient selection for TR-RYGB was completely unaffected in this series as we offered both equivalently to our patients, TR-RYGB and L-RYGB. Neither we had principal contraindications for the robotic approach when RYGB was generally indicated nor we had any positive selection criteria in this first period of the robotic program.

Prospective studies with larger patient cohorts and long-term data are necessary and might demonstrate advantages of the robotic platform for subgroups of patients and particular indications (super-obesity, revisional bariatric surgery) even under consideration of potentially longer operation times.

## Conclusion

TR-RYGB with hand-sewn gastrojejunostomy is a save procedure and can be carried out by a bariatric surgeon with experience in L-RYGB resulting in complication rates comparably low as for the laparoscopic approach. The learning curve is considerably steep and a first plateau is reached after 30 TR-RYGBs with an ongoing but less pronounced gain of experience by further procedures. However, beside its good feasibility and safety ab initio the conclusive role of TR-RYGB has to be evaluated in larger series with long-term results and higher levels of evidence.

## References

[1] Welbourn R, Hollyman M, Kinsman R, Dixon J, Liem R, Ottosson J (2019). Bariatric surgery worldwide: baseline demographic description and one-year outcomes from the fourth IFSO global registry report 2018. Obes Surg.

[2] Angrisani L, Santonicola A, Iovino P, Ramos A, Shikora S, Kow L (2021). Bariatric surgery survey 2018: similarities and disparities among the 5 IFSO chapters. Obes Surg.

[3] Schauer P, Ikramuddin S, Hamad G, Gourash W (2003). The learning curve for laparoscopic Roux-en-Y gastric bypass is 100 cases. Surg Endosc.

[4] Doumouras AG, Saleh F, Anvari S, Gmora S, Anvari M, Hong D (2018). Mastery in bariatric surgery: the long-term surgeon learning curve of Roux-en-Y gastric bypass. Ann Surg.

[5] Benizri EI, Renaud M, Reibel N, Germain A, Ziegler O, Zarnegar R (2013). Perioperative outcomes after totally robotic gastric bypass: a prospective nonrandomized controlled study. Am J Surg.

[6] Moon RC, Gutierrez JC, Royall NA, Teixeira AF, Jawad MA (2016). Robotic Roux-en-Y gastric bypass, is it safer than laparoscopic bypass?. Obes Surg.

[7] Ahmad A, Carleton JD, Ahmad ZF, Agarwala A (2016). Laparoscopic versus robotic-assisted Roux-en-Y gastric bypass: a retrospective, single-center study of early perioperative outcomes at a community hospital. Surg Endosc.

[8] Stefanidis D, Bailey SB, Kuwada T, Simms C, Gersin K (2018). Robotic gastric bypass may lead to fewer complications compared with laparoscopy. Surg Endosc.

[9] Cahais J, Lupinacci RM, Oberlin O, Goasguen N, Zuber K, Valverde A (2019). Less morbidity with robot-assisted gastric bypass surgery than with laparoscopic surgery?. Obes Surg.

[10] Dudash M, Kuhn J, Dove J, Fluck M, Horsley R, Gabrielsen J (2020). The longitudinal efficiency of robotic surgery: an MBSAQIP propensity matched 4-year comparison of robotic and laparoscopic bariatric surgery. Obes Surg.

[11] Tatarian T, Yang J, Wang J, Docimo S, Talamini M, Pryor AD (2021). Trends in the utilization and perioperative outcomes of primary robotic bariatric surgery from 2015 to 2018: a study of 46,764 patients from the MBSAQIP data registry. Surg Endosc.

[12] Acevedo E, Mazzei M, Zhao H, Lu X, Soans R, Edwards MA (2020). Outcomes in conventional laparoscopic versus robotic-assisted primary bariatric surgery: a retrospective, case-controlled study of the MBSAQIP database. Surg Endosc.

[13] Scarritt T, Hsu CH, Maegawa FB, Ayala AE, Mobily M, Ghaderi I (2021). Trends in utilization and perioperative outcomes in robotic-assisted bariatric surgery using the MBSAQIP database: A 4-year analysis. Obes Surg.

[14] Acevedo E, Mazzei M, Zhao H, Lu X, Edwards MA (2020). Outcomes in conventional laparoscopic versus robotic-assisted revisional bariatric surgery: a retrospective, case-controlled study of the MBSAQIP database. Surg Endosc.

[15] Gray KD, Moore MD, Elmously A, Bellorin O, Zarnegar R, Dakin G (2018). Perioperative outcomes of laparoscopic and robotic revisional bariatric surgery in a complex patient population. Obes Surg.

[16] Bertoni MV, Marengo M, Garofalo F, Volonte F, La Regina D, Gass M (2021). Robotic-assisted versus laparoscopic revisional bariatric surgery: a systematic review and meta-analysis on perioperative outcomes. Obes Surg.

[17] Bindal V, Gonzalez-Heredia R, Elli EF (2015). Outcomes of robot-assisted Roux-en-Y gastric bypass as a reoperative bariatric procedure. Obes Surg.

[18] Goldberg I, Yang J, Park J, Pryor AD, Docimo S, Bates AT (2019). Surgical trainee impact on bariatric surgery safety. Surg Endosc.

[19] Beckmann JH, Bernsmeier A, Kersebaum JN, Mehdorn AS, von Schonfels W, Taivankhuu T (2020). The impact of robotics in learning Roux-en-Y gastric bypass: a retrospective analysis of 214 laparoscopic and robotic procedures : robotic vs Laparoscopic RYGB. Obes Surg.

[20] Vilallonga R, Ruiz G, de Gordejuela A, Fort JM, Gonzalez O, Rodriguez-Luna MR, Roriz-Silva R (2021). Laparoscopic versus robot-assisted Roux-en-Y gastric bypass: a center of excellence for the eac-bc experience. J Laparoendosc Adv Surg Tech A.

[21] Clavien PA, Barkun J, de Oliveira ML, Vauthey JN, Dindo D, Schulick RD (2009). The Clavien-Dindo classification of surgical complications: five-year experience. Ann Surg.

[22] Dimou FM, Ackermann N, Chang SH, Freeman D, Eagon JC, Eckhouse SR (2021). Understanding the current role of robotic-assisted bariatric surgery. Obes Surg.

[23] Gray KD, Pomp A, Dakin G, Amanat S, Turnbull ZA, Samuels J (2018). Perioperative outcomes and anesthetic considerations of robotic bariatric surgery in a propensity-matched cohort of super obese and super-super obese patients. Surg Endosc.

[24] Rogula T, Koprivanac M, Janik MR, Petrosky JA, Nowacki AS, Dombrowska A (2018). Does robotic Roux-en-Y gastric bypass provide outcome advantages over standard laparoscopic approaches?. Obes Surg.

[25] Roriz-Silva R, Vilallonga R, Fort JM, Khoraki J, de Gordejuela AGR, Gonzalez O (2022). Robotic and laparoscopic Roux-en-Y gastric bypass after learning curve: 30-day and 12-month outcomes. J Robot Surg.

[26] Bendewald FP, Choi JN, Blythe LS, Selzer DJ, Ditslear JH, Mattar SG (2011). Comparison of hand-sewn, linear-stapled, and circular-stapled gastrojejunostomy in laparoscopic Roux-en-Y gastric bypass. Obes Surg.

[27] Stroh CE, Nesterov G, Weiner R, Benedix F, Knoll C, Pross M (2014). Circular versus linear versus hand-sewn gastrojejunostomy in Roux-en-Y-gastric bypass influence on weight loss and amelioration of comorbidities: data analysis from a quality assurance study of the surgical treatment of obesity in Germany. Front Surg..

[28] Hubens G, Balliu L, Ruppert M, Gypen B, Van Tu T, Vaneerdeweg W (2008). Roux-en-Y gastric bypass procedure performed with the da Vinci robot system: is it worth it?. Surg Endosc.

[29] Renaud M, Reibel N, Zarnegar R, Germain A, Quilliot D, Ayav A (2013). Multifactorial analysis of the learning curve for totally robotic Roux-en-Y gastric bypass for morbid obesity. Obes Surg.

